# Temporal Overlap and Co-Occurrence in a Guild of Sub-Tropical Tephritid Fruit Flies

**DOI:** 10.1371/journal.pone.0132124

**Published:** 2015-07-10

**Authors:** Gleidyane N. Lopes, Miguel F. Souza-Filho, Nicholas J. Gotelli, Leandro J. U. Lemos, Wesley A. C. Godoy, Roberto A. Zucchi

**Affiliations:** 1 “Departamento de Entomologia e Acarologia”, “Escola Superior de Agricultura ‘Luiz de Queiroz’–ESALQ / USP”, Piracicaba, São Paulo, Brazil; 2 “Instituto Biológico”, “Secretaria de Agricultura e Abastecimento”, Campinas, São Paulo, Brazil; 3 Department of Biology, University of Vermont, Burlington, Vermont, United States of America; University of Thessaly, GREECE

## Abstract

Studies of community assembly have emphasized snapshot comparisons of spatially replicated samples from “natural” assemblages. Agro-ecosystems are characterized by relatively little habitat heterogeneity and no dispersal barriers for actively flying insects. Therefore, dynamic patterns of species segregation and aggregation are more likely to reflect the direct or indirect effects of species interactions. We studied the temporal organization of a guild of 21 congeneric species of *Anastrepha* that colonized fruit orchards in Monte Alegre do Sul, São Paulo, Brazil. This assemblage also included the introduced Mediterranean fruit fly *Ceratitis capitata*. One hundred six consecutive weekly censuses (11 Jan 2002-16 Jan 2004) of flies in guava, loquat, and peach orchards revealed a pattern of minimum abundance during the coldest months of each year (June and July) and a maximum abundance during periods of flowering and fruit ripening. Overall, phenological overlap was greater than expected by chance. However, conditioned on the pattern of seasonal abundances, temporal occurrence and abundance matrices exhibited patterns of significant species segregation and anti-nestedness. In each year, the 3 orchards contained a small number of species pairs that exhibited statistically significant temporal segregation or aggregation. Most aggregated and segregated pairs reflected seasonal shifts in species presences that were not related to variation in air temperature. Most of the significant pairwise associations involved *C*. *capitata*: 8 of the 11 segregated pairs and 2 of the 7 aggregated pairs. These results suggest that species interactions between introduced and native species can be an important determinant of species associations in agro-ecosystems.

## Introduction

A major research focus for many decades in community ecology has been the elucidation of general assembly rules that govern the composition of local faunas [1]. These assembly rules might reflect the outcome of both positive and negative species interactions [2], habitat filtering [3], dispersal limitations [4], phylogenetic effects [5], and historical processes [4], as well as neutral or stochastic mechanisms [6].

Much of this research has been conducted with snapshot surveys of insular assemblages [7], in which islands are treated as replicates in a “natural experiment” [8]. This kind of analysis assumes that spatial variation in community structure reflects dynamic processes of colonization and extinction [9]. However, the substitution of space for time in the study of dynamics is not always warranted [10], and dynamical systems can be studied more directly by tracking changes in the species composition of an assemblage through time [1]. For example, Diamond’s 1975 [11] spatially defined “checkerboard distribution” is a pair of species that never occur together on the same island. In a dynamic system, a checkerboard distribution would correspond to a pair of species in a single site that never occur together at the same time.

A second challenge in snapshot survey of insular assemblages is that islands usually vary considerably in their area [12], habitat diversity [13], and isolation from colonization sources [14]. Moreover, the composition of many contemporary island faunas strongly reflects human-caused extinctions [15] and introductions [14]. These typical sources of spatial heterogeneity in insular systems make it challenging to document patterns of community assembly and the role of interspecific interactions.

Agro-ecosystems offer the potential to observe the temporal dynamics of species composition in a system with simplified habitat structure [16] and relatively few limits to dispersal [17]. Tropical fruit orchards represent a study system in which temporally concentrated food resources (flowers and fruits) may be the focus of resource competition among colonizing arthropods [18]. Adult fruit flies, for example, feed on nectar of flowers and other protein sources on the surface of leaves and fruit, whereas larval fruit flies feed exclusively on fruit pulp [19]. Exotic pest species are common in tropical fruit orchards [20], where they have the potential to displace the native biota [21], and to impose significant economic costs from pest control and fruit damage [22].

In this study, we examined the temporal dynamics of a guild of 22 species of tephritid fruit flies that colonized guava, loquat, and peach orchards in southern Brazil over a 2-year period. Twenty one of these species were congeners in the genus *Anastrepha*. The remaining species was the exotic Mediterranean fruit fly *Ceratitis capitata*, an important agricultural pest. We used a suite of null model tests to first ask whether there was evidence of non-random temporal covariation in the occurrence and abundance of these species. Next, we conducted a pairwise analysis to identify particular pairs of species that exhibited significant temporal aggregation or segregation. Finally, we asked whether such aggregation or segregation could be attributed to patterns of phenology or temporal variation in air temperature.

## Materials and Methods

### Study site

This research was conducted at the Experimental Station of the “Polo Regional de Desenvolvimento Tecnológico dos Agronegócios do Leste Paulista/Agência Paulista de Tecnologia dos Agronegócios” (PRDTALP/APTA) in Monte Alegre do Sul, São Paulo, Brazil (22°40’ 50” S and 46°40’ 45” W; 760 m), from 11 January 2002 to 16 January 2004. The sampling was conducted in single orchards of guava (*Psidium guajava* L.), loquat [*Eriobotrya japonica* (Thunb.) Lindl.] and peach [*Prunus persica* (L.) Batsch]. Although both loquat and peach are non-native to Brazil, they are readily colonized by both native and introduced tephritid flies [23]. The area of each orchard was 4,545.50 m^2^ (guava), 4,615.00 m^2^ (loquat) and 4,438.00 m^2^ (peach). No pesticide was applied in these orchards during the study period, and the field traps collected only agriculturally important fruit flies and did not sample any endangered or protected species.

The mean annual temperature during the two years of censusing (Jan 2002-Jan 2004) ranged from 20.8 to 20.5°C (minimum temperatures of 2 to 3.2°C and maximum temperatures of 35.2 to 34.6°C), with total annual rainfall of 1,440 to 1,358 mm (rainy period from October to March) and mean relative humidity ranging from 87.8 to 86.9. Weather data were obtained from an automated weather recording station located in PRDTALP / APTA, approximately 250 m from the orchards.

### Sampling and species identification

Fruit flies were collected in McPhail-type traps containing protein-rich torula yeast, which is a strong attractant for *Anastrepha* fruit flies. Fresh baits were deployed bi-weekly. This trap consists of two-piece plastic (a transparent upper and a yellow base invaginated to catch fruit flies). In each orchard (guava, loquat and peach), three traps were hung in the apical third of each of three fruit trees, and traps were censused weekly from 11 January 2002 to 16 January 2004. Contents of the three traps in each orchard were pooled weekly, resulting in 53 consecutive weekly samples for each year and each orchard. Because of the standardized sampling design using fresh baits, we assume that the large differences in numbers of individuals trapped of different species mostly reflect differences in the relative sizes of their populations in each orchard, rather than differential attractiveness of torula or changes through time in the relative trapping efficiency of different fruit fly species.

Trap contents were processed in the laboratory of PRDTALP / APTA. We identified specimens of the genus *Anastrepha* to the species level based on characters of the wing and thoracic mediotergite, the subscutellum, and especially the tip of the ventral aculeus. Adults of *Anastrepha* spp. and *C*. *capitata* were sexed, quantified, labeled, and stored in 70% ethanol. All analyses were based only on the abundance and identity of female flies because males of *Anastrepha* species do not exhibit morphological characters that can be reliably used for species identification. The name *A*. *fraterculus* is being used herein *sensu lato* because the *Anastrepha fraterculus* complex comprises several cryptic species [24], [25]. Voucher specimens were deposited at the Laboratório de Ecologia Econômica, Centro Experimental, Instituto Biológico, Campinas, São Paulo, Brazil.

### Niche Overlap Analyses

The data collected from each orchard in a year were organized into temporal abundance matrices in which each row represents a species, each column represents a sample, and each entry represents the proportional abundance of a species represented in a sampling period. Within a row (= species), these proportions were rescaled to sum to 100 across all of the columns (= sampling dates) for the year. This rescaling is appropriate for analyzing the abundances of all species on a common relative scale, and preventing the results from being biased towards patterns in the most common species. For each matrix, we calculated the average pairwise niche overlap among all possible pairs of species using the Pianka 1973 [26] and Czechanowski [27] niche overlap indices. These indices range between 0 for a species pair that never co-occurs in time and 1.0 for a species pair that exhibits complete temporal overlap.

We used two different null model analyses to generate the expected niche overlap assuming no species interactions and equal suitability of different time intervals. First, we used Rosario Version 1.0 [28] to model each year as a circular time interval. This null model shifts the existing distribution around the circle, preserving the observed pattern of temporal autocorrelation in the data.

We also analysed the data with the randomization algorithm RA3 in EcoSim Version 7.72 [29]. This algorithm reshuffles the proportional abundances for each species among the different time periods and is not constrained to match the observed temporal autocorrelation. For each analysis (2 years ☓ 3 orchards), we simulated 1000 null assemblages, created the histogram of simulated niche overlap values, and estimated the tail probability (one-tailed) of the observed data under the null hypothesis (p(observed niche overlap | H_0_)).

To insure that the results were not strongly influenced by sampling noise for rare species, we re-ran all null model analyses (niche overlap and co-occurrence) using only the 7 most common species from the collections. Results were qualitatively similar to the analyses of the complete samples that we present here.

### Co-occurrence Analyses

We analysed species co-occurrence patterns using both the original abundance data (counts of the abundance of each species in a sample) and incidence data (presence or absence of each species in a sample). The abundance data were analyzed with the program CoOccurrence Version 2.0 [30]. We used the IT null model, which resamples the matrix cells proportional to observed row and column totals until the total row and column abundances are achieved [31]. Abundance patterns of co-occurrence were quantified with the “checker” and “anodf” indices. These indices are analogous to measures used in presence-absence analysis, and quantify respectively the number of checkerboard units, and the degree of nestedness in the abundance matrix [31].

The data from each orchard collected in a year were also organized into binary presence-absence matrices in which each row represents a species, each column represents a sample, and entries indicate the presence (1) or absence (0) of a species at a sampling period [32]. For each matrix, the co-occurrence pattern for each unique species pair was summarized as the C score [33], which measures the degree of aggregation (low C-score) or segregation (high C-score) for a species pair.

The statistical significance for each species pair was calculated with the software package PAIRS [34], using the fixed-fixed null model. This algorithm randomizes the occurrence of presences and absences, but preserves the row and column sums of the original matrix, and has good statistical properties [35]. Because row totals are preserved, inherent differences in the commonness and rarity of species are incorporated into the analysis. Because the column totals are preserved, inherent differences in the suitability of different time periods (due possibly to differences in temperature, humidity, or other common environmental effects) are also incorporated into the analysis. In some cases, these procedures may be overly conservative, but they prevent many Type I statistical errors (incorrectly rejecting a true null hypothesis), which is important for the analysis of non-experimental data. See Gotelli and Ulrich 2012 [36] for additional discussion.

In our analysis, with *n* species in the matrix, there are (*n*)(*n*-1)/2 possible pairs of species, and many of these pairs may not be biologically or statistically independent from each other [37]. The PAIRS software adjusts for the large number of pairwise significance tests in the analysis. We used the Empirical Bayes Confidence Limit method in PAIRS to adjust the *p*-values for each pair of species [34]. As in any statistical analysis, it is difficult to detect significance if both members of a species pair are very rare.

Finally, once statistically significant pairs of aggregated or segregated species were identified, we carried out additional tests [38] to determine whether the pattern of aggregation or segregation could be attributed to seasonal or thermal associations. For each segregated pair, we compared with a t-test the calendar (Julian) date and the air temperature during sampling of samples that contained one of the two species (10) to samples that contained the other species (01). If the Julian dates were statistically different for these two groups of samples, the species pair was segregated by season. Within each set of yearly samples, we analysed the average sampling dates as circular data, using the ‘aov.circular’ function in the R package ‘circular’. This analysis ensures that average dates are calculated correctly for samples that may overlap seasonally from December and January. If the air temperatures were statistically different for samples that contained each species occurring by itself, the species pair was segregated by temperature. If neither temperature nor Julian date were significantly different, the species pair was segregated in time, but the presence and absence sequence formed a temporal checkerboard that could not be attributed to seasonality or temperature differences.

**Fig 1:**
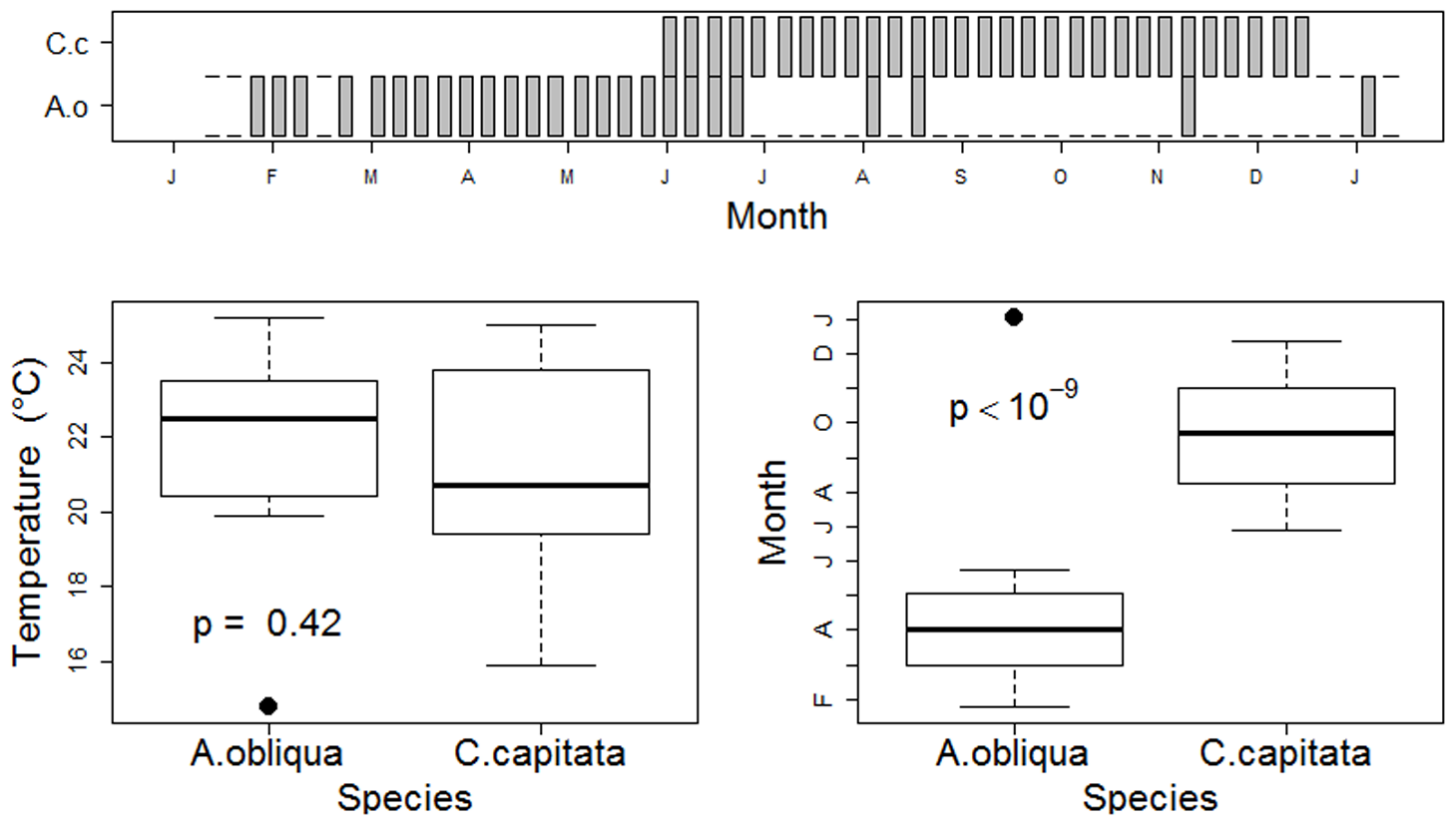
Co-occurrence pattern based on presence-absence data of a significantly segregated species pair, *A*. *obliqua* ☓ *C*. *capitata*. The upper panel illustrates the co-occurrence patterns in temporal samples of *A*. *obliqua* (unfilled bars) and *C*. *capitata* (grey bars) collected in a loquat orchard in 2002. Box plots illustrate average air temperature (°C) and Julian collection date (plotted on a monthly scale) for samples that contained only *A*. *obliqua* or only *C*. *capitata* (temporal checkerboards). This species pair is segregated on the basis of seasonality, but not on the basis of temperature.

For each aggregated pair, we carried out an analogous set of calculations, but this time comparing the calendar (Julian) date and the temperature of samples that contained both species (11) with those that contained neither species (00). These analyses revealed whether species aggregations could be attributed to seasonality or similar temperature affinities. Data for the association of *A*. *obliqua* and *C*. *capitata* in the loquat orchard illustrate how measures of air temperature and sampling date were analyzed for each significant species pair (Fig 1). In this example, the two species were collected at similar air temperatures throughout the year (temperature test; *p* = 0.42), but once *C*. *capitata* began to occur in the second half of the year, the occurrence of *A*. *obliqua* was greatly reduced (seasonality test; *p* < 10^−9^).

### Data Sources and Graphics

Original data matrices for all analyses are given in S1a–S1c Table. All graphs were created with R Version 3.0.1 [39], and R script files for the graphs are given in S1 Text.

## Results

For the guava, loquat, and peach orchards sampled in 2002 and 2003, we collected 106 samples, and captured 25,872 individuals of the exotic Mediterranean fruit fly *Ceratitis capitata* (21,252 females and 4,620 males) and 89,958 individuals of 21 congeneric species of the genus *Anastrepha* (48,041 females and 41,916 males; Table 1).

**Table 1:** Total females abundance of native tephritid *Anastrepha* species and the introduced Mediterranean fruit fly *Ceratitis capitata* collected in three fruit orchards, Monte Alegre do Sul, São Paulo, Brazil (2002–2003).

Species	Guava	Loquat	Peach
*A*. *fraterculus* (Wied.)	14,414	27,792	2,685
*A*. *sororcula* Zucchi	69	486	190
*A*. *bistrigata* Bezzi	803	534	43
*A*. *obliqua* (Macquart)	488	371	19
*A*. *grandis* (Macquart)	5	32	7
*A*. *pseudoparallela* (Loew)	2	19	1
*A*. *bahiensis* Lima	2	33	0
*A*. *barbiellinii* Lima	2	6	0
*A*. *consobrina* (Loew)	0	3	0
*A*. *distincta* Lima	4	5	0
*A*. *serpentina* (Wied.)	0	4	0
*A*. *montei* Lima	2	1	0
*A*. *punctata* Hendel	0	1	0
*A*. *amita* Zucchi	0	1	0
*A*. *leptozona* Hendel	0	1	0
*A*. *dissimilis* Stone	0	1	0
*A*. *daciformis* Bezzi	0	1	0
*A*. *zenildae* Zucchi	2	5	0
*A*. *turpiniae* Stone	0	3	0
*A*. *pickeli* Lima	3	0	0
*A*. *elegans* Blanchard	1	0	0
*C*. *capitata* (Wied.)	3,556	8,449	9,247

**Fig 2:**
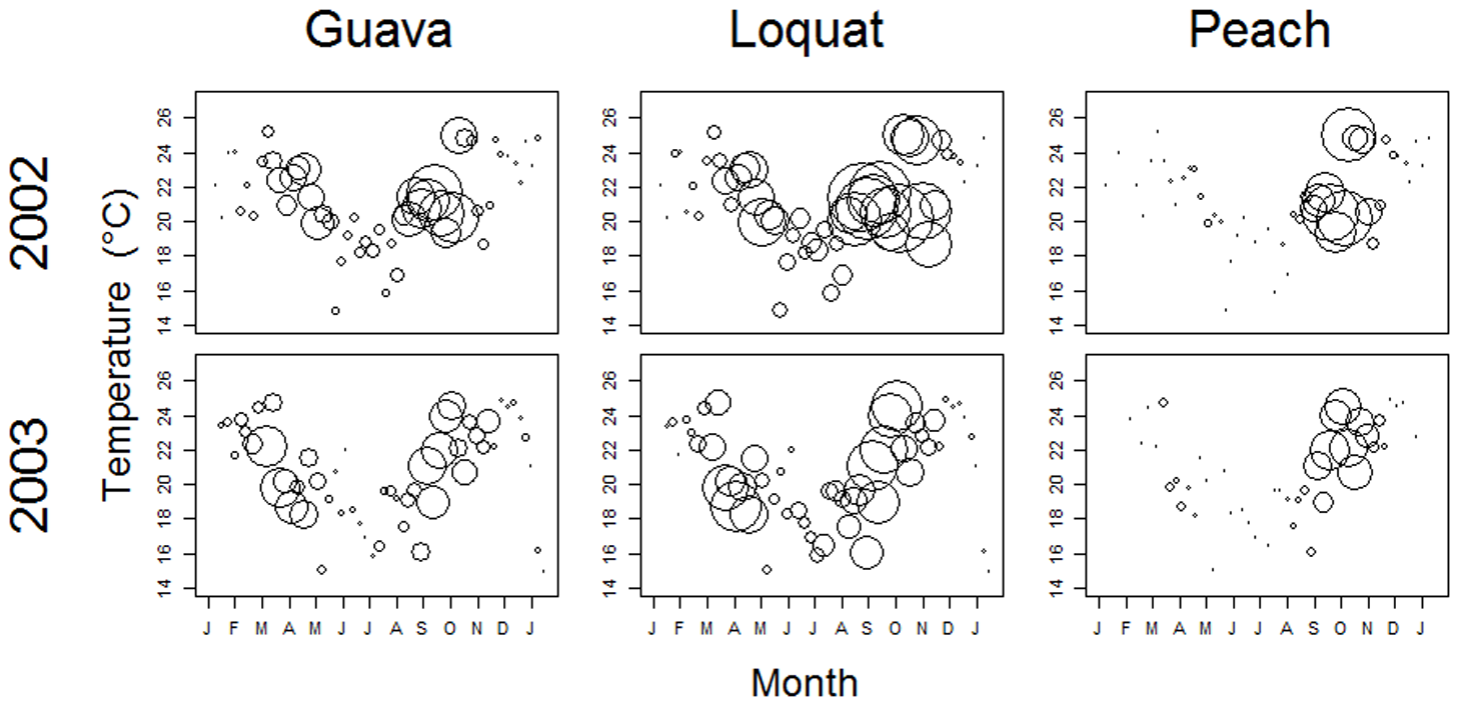
Fruit fly abundance, air temperature (°C), and sampling date for 3 fruit orchards sampled in Monte Alegre do Sul, São Paulo, Brazil in 2002 and 2003. The area of each circle is proportional to the total abundance of fruit flies collected in the sample.

**Fig 3:**
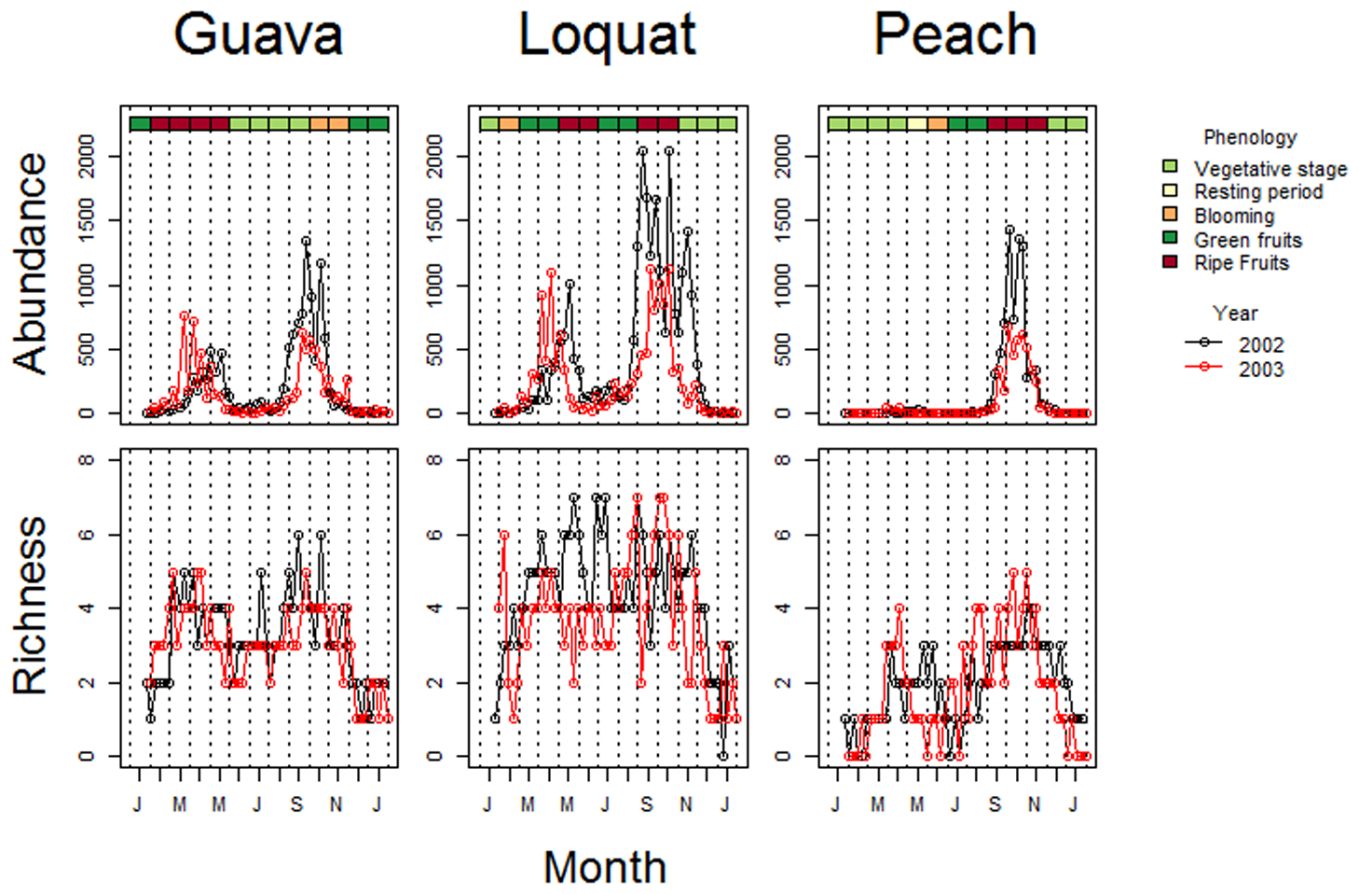
Total abundance and species richness data of fruit flies in relation to time and fruit tree phenology for samples collected during two years (2002–2003) in an experimental station in Monte Alegre do Sul, state of São Paulo, Brazil.

In all orchards, air temperatures reached minima during June and July, which also corresponded to low capture rates of flies in traps (Fig 2). Each year, fruit fly abundance reached a peak as green fruit ripened, although on guava trees, fruit fly abundance was also high when guava fruits were not available (Fig 3). In each orchard, the number of species of fruit flies recorded varied between the two years: 11 and 12 species in guava; 18 and 13 species in loquat, and 7 and 6 species in peach. However, fruit fly species richness did not exhibit any distinct phenological pattern or peaks that corresponded with fruit availability (Fig 3).

### Niche Overlap Patterns

Compared to both the Rosario and the EcoSim null models, average pairwise niche overlap in abundance was significantly greater than expected by chance for assemblages in all 3 orchards measured in each of the two years (Table 2). Results did not differ using either the Czekanowski or the Pianka niche overlap indices.

**Table 2:** Observed and expected temporal niche overlap (Pianka’s index) and the one-tailed *p*-value based on 1000 randomizations for fruit fly assemblages sampled in three orchard types over two years.

Orchard/Year	Observed overlap	Expected overlap	*p* value
Guava/2002	0.20304	0.11051	<0.001
Guava/2003	0.14721	0.07252	0.001
Loquat/2002	0.12844	0.09959	0.005
Loquat/2003	0.20217	0.11595	<0.001
Peach/2002	0.16114	0.09668	0.024
Peach/2003	0.36424	0.12663	<0.001

### Species Co-occurrence Patterns

For both presence absence and abundance data, community wide patterns were consistent among orchards and across years: on average, species pairs were more segregated than expected by chance (large positive C-scores), and the matrices showed overall patterns of anti-nestedness (large negative nodf scores; Table 3).

**Table 3:** Z-scores for null model analyses of presence-absence and abundance matrices of fruit flies in 3 orchards.

	Presence-absence matrix		Abundance matrix	
Orchard/Year	C-score	NODF	Checker	Anodf
Guava/2002	3.2	-2.18	5.84	-2.19
Guava/2003	4.74	-2.21	6.5	-3.64
Loquat/2002	8.47	-8.23	11.96	-8.07
Loquat/2003	3.6	-2.42	4.96	-4.85
Peach/2002	3.42	-3.10	4.05	-3.51
Peach/2003	4.20	-2.38	5.64	-2.76

The C-score index for presence-absence and the Checker index for abundance measure species segregation and aggregation. The NODF index for presence-absence and the Anodf index for abundance measure nestedness and anti-nestedness. Z scores larger than |2| indicate statistical significance. Positive values for the C-score and Checker indices indicate species segregation, whereas negative values for NODF and Anodf indicate anti-nestedness patterns. The fixed-fixed null model was used to randomize the presence-absence matrices, whereas the IT null model was used to randomize the abundance matrices. See Ulrich and Gotelli 2010 [31] for details.

For co-occurrence analysis of individual species pairs, there was a total of 388 possible species pairs that could be formed from the data matrices. Of these 388, 18 pairs (11 segregated and 7 aggregated) were statistically significant using the empirical Bayes confidence limit criterion (Table 4).

**Table 4:** Significantly aggregated and segregated species detected in species co-occurrence analysis.

	Guava		Loquat		Peach	
Species pairs	2002	2003	2002	2003	2002	2003
*C*. *capitata* x *A*. *obliqua*	-	-	-	-		
*C*. *capitata* x *A*. *grandis*	-		-			
*A*. *obliqua* x *A*. *distincta*	+					
*A*. *obliqua* x *A*. *sororcula*		+		+		
*A*. *obliqua* x *A*. *grandis*			+			
*A*. *bistrigata* x *A*. *sororcula*			+			
*A*. *bistrigata* x *A*. *pseudoparallela*			-			
*A*. *sororcula* x *A*. *pseudoparallela*			-			
*A*. *obliqua* x *A*. *pseudoparallela*				-		
*C*. *capitata* x *A*. *bistrigata*					-	
*C*. *capitata* x *A*. *sororcula*		-			+	+

**Legend**:

- = Segregated

+ = Aggregated

□ = Pair not formed

- = significantly segregated species pair; + = significantly aggregated species pair; white = pair not occurring in a particular orchard/year. Pairwise analyses control for false discovery rates using the methods in Gotelli and Ulrich 2010 [37].

**Fig 4:**
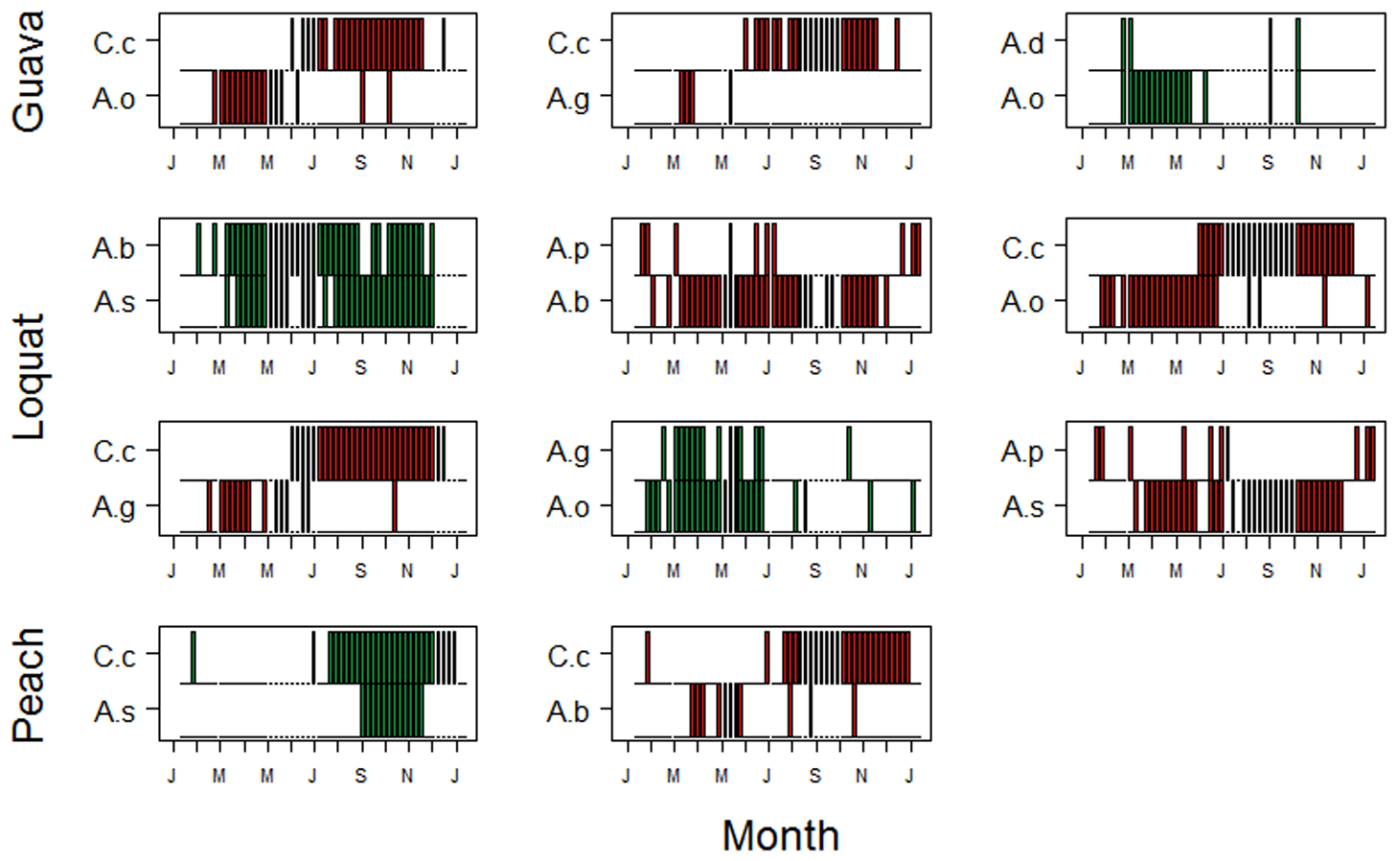
Co-occurrence pattern based on presence-absence data of significantly segregated (red panels) and aggregated (green panels) pairs of tephritid fruit flies sampled in 3 orchards in the year 2002. Each bar represents the presence of each species in a particular sample. Species abbreviations are: A.b. = *A*. *bistrigata*; A.d. = *A*. *distincta*; A.g. = *A*. *grandis*; A.o. = *A*. *obliqua*; A.p. = *A*. *pseudoparallela*; A.s. = *A*. *sororcula*; C.c. = *C*. *capitata*.

**Fig 5:**
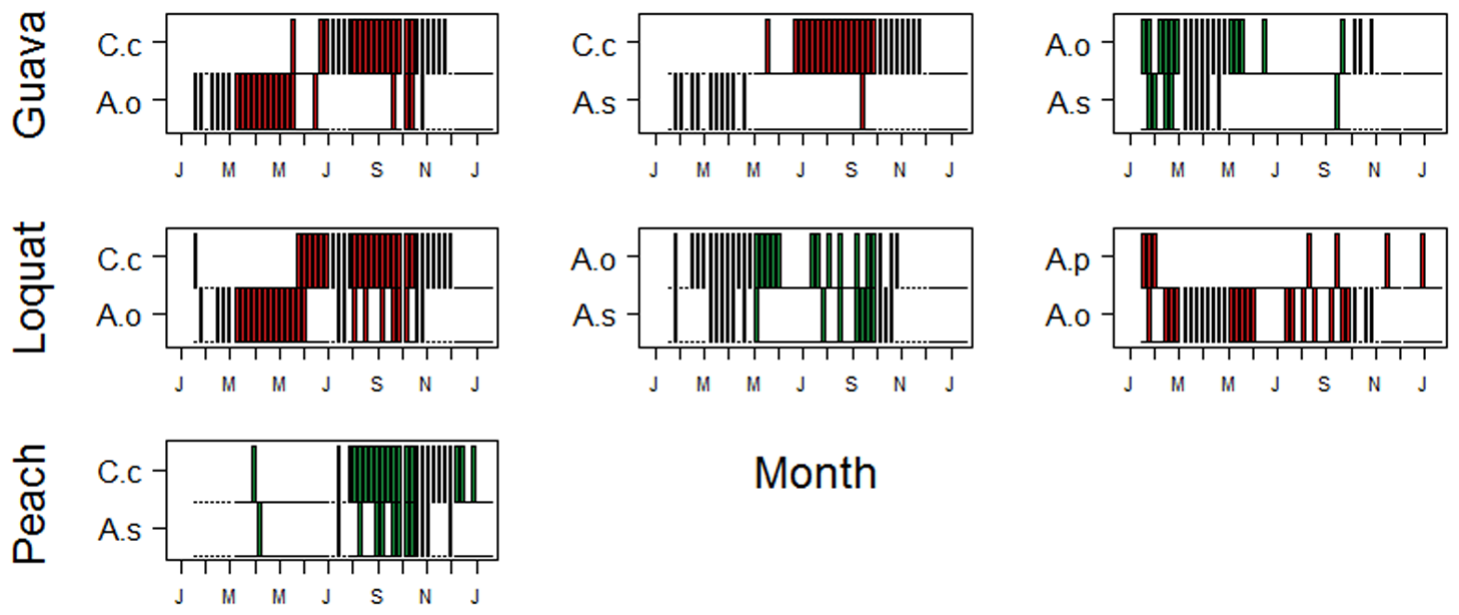
Co-occurrence pattern based on presence-absence data of significantly segregated (red panels) and aggregated (green panels) pairs of tephritid fruit flies sampled in 3 orchards in the year 2003. Abbreviations as in Fig 4.

Figs 4 and 5 illustrate the temporal pattern of the 18 species pairs with significant positive or negative associations. In the guava orchard in first year, there were three non-random pairs of species, of which two were segregated (*C*. *capitata* x *A*. *obliqua* and *C*. *capitata* x *A*. *grandis*) and one was aggregated (*A*. *obliqua* x *A*. *distincta*). In the second year, the pattern was very similar, with two pairs of segregated species (*C*. *capitata* x *A*. *obliqua* and *C*. *capitata* x *A*. *sororcula*) and one pair of aggregated species (*A*. *obliqua* x *A*. *sororcula*).

In the loquat orchard in the first year, there were four significantly segregated species pairs (*C*. *capitata* x *A*. *obliqua*, *C*. *capitata* x *A*. *grandis*, *A*. *bistrigata* x *A*. *pseudoparallela* and *A*. *sororcula* x *A*. *pseudoparallela*) and two aggregated species pairs (*A*. *sororcula* x *A*. *bistrigata* and *A*. *obliqua* x *A*. *grandis*). In the second year, there were two segregated pairs (*C*. *capitata* x *A*. *obliqua* and *A*. *obliqua* x *A*. *pseudoparallela*) and one aggregated pair (*A*. *obliqua* x *A*. *sororcula*). In the peach orchard in the first year, there were two segregated species pairs (*C*. *capitata* x *A*. *bistrigata*) and one aggregated pair (*C*. *capitata* x *A*. *sororcula*). In the second year, there was only one aggregated pair (*C*. *capitata* x *A*. *sororcula*).

Among these 18 significant pairs, 13 pairs were segregated or aggregated by season (differences in average Julian date), and 1 pair was aggregated on the basis of average air temperature. Four pairs were statistically non-random in co-occurrence, but the pattern was not related to associations with season or air temperature (Table 5).

**Table 5:** Analysis of average air temperatures and Julian calendar dates (= season) for time periods of species presences and absences in statistically significant species aggregations and segregations (see Table 4).

	Guava		Loquat		Peach	
**Aggregated species pairs**	2002	2003	2002	2003	2002	2003
*A*. *obliqua* x *A*. *distincta*	\					
*A*. *obliqua* x *A*. *sororcula*		↔		↔		
*A*. *obliqua* x *A*. *grandis*			↔			
*A*. *bistrigata* x *A*. *sororcula*			Δ			
*C*. *capitata* x *A*. *sororcula*					↔	↔
**Segregated species pairs**						
*C*. *capitata* x *A*. *sororcula*		↔				
*C*. *capitata* x *A*. *obliqua*	↔	↔	↔	↔		
*C*. *capitata* x *A*. *grandis*	↔		↔			
*C*. *capitata* x *A*. *bistrigata*					↔	
*A*. *bistrigata* x *A*. *pseudoparallela*			\			
*A*. *sororcula* x *A*. *pseudoparallela*			\			
*A*. *obliqua* x *A*. *pseudoparallela*				\		

**Legend**:

Temp. Season

↔ = N.S. S.

Δ = S. N.S.

\ = N.S. N.S.

□ = Species pair association N.S.

S. = Significant

N.S. = No significant

Significant results indicate a pair of species in which aggregation or segregation was associated with non-random patterns of air temperature or seasonality (see Fig 1). ↔ = significant seasonal difference, no temperature difference; Δ = significant temperature difference, no seasonal difference; \ = significant species aggregation or segregation without associated differences in air temperature or seasonality; white = segregation or aggregation not statistically significant.

## Discussion

Fruit fly abundance is strongly correlated with temperature and abiotic factors [40] and the availability of habitat, food, water, and oviposition substrates [41], which influence the mobility of fruit flies [40]. *Anastrepha elegans* was the only one of the six rare species (Table 1) collected in the guava orchard, probably because it uses guava as host [42]. Although *Anastrepha punctata*, *A*. *amita*, *A*. *leptozona*, *A*. *dissimilis* and *A*. *daciformis* were collected in loquat orchard, these species have never been recorded in loquat. Apparently, the loquat orchard provides best conditions for these species. Air temperature and fruit fly abundance in all 3 orchards were at an annual minimum in June and July (Fig 2), whereas peaks of fly abundance seem to be more correlated with the availability of green or ripe fruit (Fig 3). Both patterns are consistent with the null model analysis, which revealed phenological clumping of abundance (Table 3). However, it is noteworthy that temporal variation in species richness does not follow any seasonal or phenological trend (Fig 3), perhaps reflecting the non-random co-occurrence patterns of many species pairs within the assemblages.

Most species pairs consisted of congeners of the 21 species of *Anastrepha*, so it is striking how many of the significant pairwise associations involved the single pest species *Ceratitis*. Based on the frequencies of different species in each orchard and each year, the expected number of significant associations between *Ceratitis* and a species of *Anastrepha* should have been 2.55, whereas the observed number was 10 (X^2^_1_ = 19.56, *p* = 9.7 x 10^−6^).

Although there was a large number of non-random associations of *Ceratitis* with native species of *Anastrepha*, to our knowledge there is no study clearly demonstrating that *C*. *capitata* displaces *Anastrepha* species. However, previous studies have demonstrated interference competition between adult tephritids, and both inter-specific interference and exploitation competition between tephritid larvae (see Duyck *et al*. 2004 [43] for review). Because the orchards represented relatively homogeneous habitat for fruit flies with few or no barriers to dispersal, non-random co-occurrence patterns among different species pairs are likely to reflect direct and indirect species interactions. Direct competitive interactions are likely to generate negative co-occurrence patterns, but indirect competitive effects involving 3 or more species may lead to some positive pairwise associations. Parasitoids were not censused in this study, but they may also lead to positive or negative associations between fruit fly species. Five Braconidae parositoid species (*Asobara anastrephae*, *Doryctobracon areolatus*, *D*. *brasiliensis*, *Opius bellus* and *Utetes anastrephae*) and three Figitidae parasitoid species (*Aganaspis pelleranoi*, *Lopheucoila anastrephae* and *Trybliographa infuscate*) attacking fruit flies on guava and peach fruits were collected by Souza-Filho *et al*. 2009 [44], so there is the potential for complex trophic interactions in this system.

In two other null model analyses, the co-occurrence of native ant species was more random in the presence of an invading species than in its absence [45], [46]. It would be interesting to analyze the co-occurrence structure of the *Anastrepha* guild in the absence of *C*. *capitata*. However, it would be difficult to find such assemblages, because *Ceratitis* has invaded most fruit orchards in Brazil since 1901 [47], [48] and it is still expanding with recent invasions of northern Brazil [49].

The common pairs of aggregated interactions were *A*. *obliqua* x *A*. *sororcula* (2) and *C*. *capitata* x *A*. *sororcula* (2), followed by *A*. *obliqua* x *A*. *distincta* (1), *A*. *obliqua* x *A*. *grandis* (1), *A*. *bistrigata* x *A*. *sororcula* (1) (Figs 4 and 5). The only positive interactions with *C*. *capitata* were in the peach orchard, where few species were collected and it is preferential host of this fruit fly specie [42]. These associations could reflect facilitative interactions, indirect effects with other species, or associations based on use of critical unmeasured resources. *Anastrepha sororcula*, *A*. *bistrigata* and *A*. *obliqua* prefer fruits of Myrtaceae [42] and they were also collected in fruits of guava during this study [44]. Fruit flies species began to attack fruits of guava, loquat and peach in the intermediate ripening stage of fruits and the population of flies showed an increase tendency when full fruits were completely ripe [44]. *Anastrepha grandis* is a quarantine species, which attacks melon and cucurbits, and *A*. *distincta* is a minor pest, which develops in Fabaceae species that are not of economic importance.

It is important to note that the co-occurrence analyses, by definition, depends on covariation of species presences and absences. For this reason, the widespread polyphagous *A*. *fraterculus* did not exhibit any statistical associations because it was present in all samples in this study due to host succession [50].

In summary, understanding the causes of species associations in spatially replicated “natural experiments” is challenging because habitat associations and dispersal limitation as well as species interactions can lead to non-random co-occurrence patterns [38]. Temporal patterns of co-occurring fruit flies represent a system in which dispersal limitations and habitat variation are minimal, so that patterns of species aggregation and segregation are more likely to represent the direct and indirect effects of species interactions.

## Supporting Information

S1 Table Original data matrices for all analyses of fruit flies species collected in orchards of (a) loquat, (b) peach and (c) guava, Monte Alegre do Sul, São Paulo, Brazil (2002–2003). (XLS)

S1 Text R script files for the graphs. (TXT)
